# Do different lipid components accelerate the pathogenesis and severity of Diabetic Retinopathy?

**DOI:** 10.1186/s40942-022-00390-4

**Published:** 2022-06-11

**Authors:** Lakshmi Kanta Mondal, Subhasish Pramanik, Subhankar Chowdhury, Chiranjit Bose, Debgopal Bera, Ayindrila Saha, Koena Bhattacharjee

**Affiliations:** 1 Department of Endocrinology & Metabolism, Institute of Post Graduate Medical Education & Research and SSKM Hospital, Kolkata, 700020 West Bengal India; 2 Department of Ophthalmology, Regional Institute of Ophthalmology, Medical College Campus, Kolkata, 700 073 West Bengal India; 3 Department of Rheumatology, Institute of Post Graduate Medical Education & Research and SSKM Hospital, Kolkata, 700020 West Bengal India

**Keywords:** Type 2 diabetes mellitus, Diabetic retinopathy, Lipid peroxides, Advanced lipoxidation end products, Vascular endothelial growth factor

## Abstract

**Background:**

To assess the association of lipid and lipid-derived toxic molecules in pathogenesis and severity of diabetic retinopathy (DR) in type 2 diabetes mellitus (T2DM).

**Methods:**

The present cross-sectional study included 14 healthy individuals (HC) without T2DM, 22 T2DM subjects without DR (DNR), 24 T2DM subjects with mild non-proliferative DR (MNPDR), and 24 T2DM subjects with high-risk proliferative DR (HRPDR). All subjects underwent plasma and vitreous analysis for estimation of total lipid (TL), free fatty acid (FFA), lipid peroxides (LPOs) like malondialdehyde (MDA), 4-Hydroxy-noneal (HNE), the advanced lipoxidation end product (ALE) like Hexanoyl-lysine (HLY) and vascular endothelial growth factor (VEGF) following standard spectrophotometric and enzyme-linked immunosorbent assay (ELISA) methods respectively.

**Results:**

The concentration of TL, FFA, markers of lipid peroxidation and lipoxidation as well as VEGF in plasma and vitreous were found to be significantly elevated stepwise inT2DM subjects (HRPDR > MNPDR > DNR) compared to healthy controls (HC).Further, plasma conventional lipid components like total cholesterol (TCH), low density lipoprotein cholesterol (LDL-C) and triglycerides (TG), FFA and TL showed their significant positive correlations with vitreous level of different LPOs, ALE and VEGF in the DR group.

**Conclusion:**

Total lipid and lipid-derived detrimental biomolecules ultimately result in increased secretion of VEGF and thus not only add as associated mediators in the pathogenesis of DR, these also accelerate the severity of microangiopathy in T2DM.

## Introduction

Diabetic retinopathy (DR) is the most common multifactor-associated microvascular complication of the retina and also one of the leading causes of visual impairment among individuals with diabetes in their working age group [1]. In India, the prevalence of any type of DR is 15.4% consisting of 14.7% non- sight threatening DR and 6.7% sight threatening DR [2].

The pathophysiology of DR has been extensively studied and many contributing biochemical pathways have been identified. Mostly all clinical trials and biochemical studies have demonstrated existence of strong relationship between chronic and poorly controlled glycaemia-mediated biochemical anomalies with the development and progression of DR [3–6].

Role of dyslipidaemia is not well understood yet in the pathogenesis DR [7]. A few cross sectional studies have shown a positive association between plasma conventional lipid components like Total Cholesterol (TCH), Triglycerides (TG), Low density lipoprotein cholesterol (LDL-C) and severity of DR [1, 8, 9]. Some literature has also demonstrated a link between dyslipidemia and an increase in free fatty acids (FFA) level which results in endothelial dysfunction, and apoptosis of endothelial cells. However, there is very little documentation that has established specific links between elevated FFA and the occurrence of DR [10, 11].

Recently, some researchers are also drawing the attention towards the implication of lipid peroxidation and lipoxidation in the pathogenesis and severity of DR. The retina has an unavoidable vulnerability to developing oxidative stress that results from light exposure, high oxygen consumption, continuous phagocytosis of outer segments of rods, and dense concentration of polyunsaturated fatty acids in photoreceptors [12]. One of the unpleasant effects of uncontrolled oxidative stress which is defined as an imbalance between prooxidant and antioxidant levels in the retina is cellular injury due to oxidative damage. It has long been evidenced that a high level of free radicals or reactive oxygen species generated from the metabolism of molecular oxygen can impose direct damage to lipids, especially polyunsaturated lipids containing carbon–carbon double bonds [13]. This process described as lipid peroxidation results in the formation of peroxyl free radical, lipid hydroperoxide, and some secondary by products like malondialdehyde (MDA), propanal, hexanal, and 4-hydroxynonenal (4-HNE). MDA appears to be the most mutagenic whereas 4-HNE is the most toxic to cells [14, 15]. These electrophilic aldehydes 4-HNE and di-aldehydes MDA are recognized as reactive carbonyl species (RCS) containing carbonyl moiety and chemical reactivity and react with different nucleophilic substrates like basic amino acids of proteins, deoxyribonucleic acid (DNA), and membrane phospholipids to produce advanced lipoxidation end products (ALEs) [16]. So, ALEs are defined as adducts and cross-links that are made by the reaction of produced reactive carbonyl species in lipid peroxidation with cellular proteins, DNA, and amino phospholipids in a nonenzymatic process. The consequent loss of function and structural integrity of these modified biomolecules can express a wide range of cellular dysfunctions and tissue damage.

It has been shown that ALEs can also modify function of protein by covalent bonding with the catalytic site. Role of ALEs have been proved in the proliferation of vascular smooth muscle cells, stimulation and aggregation of platelets, stimulation of different growth factors, and reduction of nitric oxide bioavailability [17, 18]. Under the background of chronic hyperglycemia, lipid peroxidation, and advanced lipoxidation end products formation, the pathways of inflammation and coagulation in the capillary beds of retina produce hypoxia which ultimate result in up-regulation of VEGF secretion, the principal driver of development and progression of DR [17, 19, 20].

In this study, we attempted to demonstrate the correlation of LPOs and ALEs with VEGF expression and also their association with the onset and severity of DR. Along with systemic estimation of this lipid and lipid derived molecules, we performed vitreous analysis for these mediators to highlight localized retinal cell expression in this disease process.

## Methods

A number of 24 subjects with high-risk proliferative diabetic retinopathy (HRPDR), 24 subjects with mild non-proliferative DR (MNPDR), 22 age and gender-matched diabetic subjects without clinically evident retinopathy (DNR), and 14 non-diabetic healthy individuals (HCs), whose clinical condition independently indicated for vitrectomy were enrolled in the present cross-sectional study. Subjects with uncontrolled hyperglycemia [either FPG and PPG level > 160 mg/dl and 250 mg/dl respectively or HbA1c (%) > 9.5] known coronary artery disease, hypertension, (systolic BP > 140 mm Hg and or diastolic BP > 90 mm Hg, or on antihypertensive medication), neuropathy (as evaluated by Michigan Neuropathy Screening Instrument), nephropathy (serum creatinine level > 1.5 mg/dl and or urinary albumin creatinine ratio 300 μg/mg), severe deficiency of B vitamins (such as thiamin, folic acid, cobalamin) any other ocular diseases (glaucoma, cataract, optic neuropathy, branch retinal vein occlusion, and Eales disease) were excluded from the present study. The subjects were chosen consecutively from the ‘Retina Clinic’ of ‘Regional Institute of Ophthalmology, Calcutta Medical College, and Kolkata, India. The study was approved by the institutional ethical committee(Ethics Committee of Regional Institute of Ophthalmology, Medical College, Kolkata, Ref No: MC/ KOL/ IEC/ NON- SPON/ 181/ 12- 2018) and informed consent was collected from all the patients according to the declaration of Helsinki.Patients with type 2 DM were diagnosed based on the guideline of the American Diabetes Association (2010). Glycaemic status was assessed by fasting plasma glucose (FPG), postprandial plasma glucose (PPG), and glycated hemoglobin (HbA1c %) level. None of the enrolled subjects in this study were taking insulin or lipid-lowering drugs during the period of study.

### Comprehensive ophthalmological examinations

All the study subjects had undergone detailed ophthalmological examinations which included slit-lamp biomicroscopy (by ± 90 diopters and Goldman 3 mirror lens), seven fields digital fundus photography with fluorescein angiography only in diabetic subjects with suspected retinopathyto be confirmed, and spectral-domain optical coherence tomography (SD-OCT) to detect macular edema. Visual functions were also evaluated by measuring visual acuity (VA). The subjects with DR were diagnosed according to the modified guideline of ‘Early Treatment of Diabetic Retinopathy Study’ (ETDRS), (1991) [21].

### Collection of blood and vitreous sample

After overnight fast 5 ml of the venous blood sample was collected from each study subject in ethylenediaminetetraacetic acid (EDTA) vacutainers. Then samples were centrifuged at 3000 r.p.m for 10 min to separate plasma from cellular components. The plasma samples were collected in cryotubes and preserved in the − 80 º C for further assessment of different biochemical parameters. On the other hand the vitreous samples from study subjects were drawn by 3-port parsplana-vitrectomy during surgery of vitreous haemorrhage, of idiopathic macular hole or removal of a dropped nucleus which occurred accidentally after blunt trauma. Vitreous was also collected during management of per-operative complications of phacoemulsification in HCs, DNR and MNPDR. The HRPDR subjects underwent vitrectomy due persistent vitreous haemorrhage. Indications and number of subjects who underwent vitrectomy have been clearly mentioned in the Table 1. Undiluted vitreous gel (500 µL) was excised from mid-vitreous by vitreous cutter and carefully aspirated into the hand-held sterile syringe attached to the suction port of the vitrectomy probe. Immediately after collection, the vitreous samples were taken in micro centrifuged tube and centrifuged at 3000 rpm for 5 min. The clear solution without any precipitate was then collected in another tube and preserved in − 80º C for further use.

### Measurement of blood glycated haemoglobin (HbA1c %) plasma glucose, and conventional lipid profile components

Blood level of HbA1C, plasma glucose (fasting and postprandial), and conventional lipid profile components were measured using commercial kits by standard manufacturer instructed protocols as discussed in our previous paper [22].

### Estimation of total lipid (TL) in plasma and vitreous

Total lipid content of plasma and vitreous samples was measured according to the modified method as proposed by Fringes and Dunn, 1970 [23]. Briefly, plasma or vitreous was mixed with concentrated sulphuric acid and incubated in a boiling water bath for 10 min. Then sulphated sample was added to the phosphoric acid in another tube. Then the tube was shaken and incubated at 37 °C temperature for 15 min. After the tube was cooled at room temperature. Optical density was measured against blank at 540 nm using a spectrophotometer. 0.6% of vanillin was treated in the same manner as vitreous and used as standard. The value was expressed in mg/dl.

### Estimation of free fatty acids (FFA) in plasma and vitreous

The level/concentration of FFA in the plasma or vitreous fluid was estimated as per protocol reported by Meeran et al. 2020 [24]. To 0.5 mL of the dried lipid fraction/0.2 ml of plasma or vitreous gel, exactly 1.0 mL of phosphate buffer (pH 6.3), 2.5 mL of copper reagent, and 6.0 ml of extraction solvent were added. The tubes were shaken well for 90 s and kept aside for 15 min. After centrifugation, 3.0 ml of the upper layer was mixed with 0.5 mL of diphenylcarbazide solution. The absorbance was read after 15 min at 550 nm. Phosphate buffer was used as the blank.

### Estimation of malondialdehyde (MDA) in plasma and vitreous

The MDA levels in the plasma and vitreous were measured by the thiobarbituric acid (TBA) assay method. In the assay procedure, MDA reacts with TBA and forms a chromogenic adduct, which was measured spectrophotometrically at 532 nm; the results were expressed as mM/L [25].

### Measurement of HNE in plasma and vitreous

Human plasma and vitreous level of HNE was estimated by competitive inhibition enzyme immunoassay technique using CUSABIO kit (cat no: CSB-E16214h) and a microplate reader (MerilyzerEiaquant, Meril Diagnostics Pvt Ltd, Vapi, Gujarat) manufacturer instructed protocol.

### Measurement of Hexanoyl lysine (HLY) in plasma and vitreous

The plasma and vitreous HLY level was measured by the competitive enzyme-linked immune sorbent assay (ELISA) method using ‘Mybiosource’ kit (cat no: MBS753480) and a microplate reader (MerilyzerEiaquant, Meril Diagnostics Pvt Ltd, Vapi, Gujarat) manufacturer instructed protocol.

### Measurement of VEGF concentration in plasma and vitreous

Human plasma and vitreous VEGF level was estimated by enzyme-linked immune sorbent assay (ELISA) method using Ray Biotech kit (cat no: ELH-VEGF-001, Norcross USA) and a microplate reader (MerilyzerEiaquant, Meril Diagnostics Pvt Ltd, Vapi, Gujarat) manufacturer instructed protocol.

### Statistical analyses

The ‘Kolmogorov–Smirnov test’ was performed to assess the assumption of normality for the data, obtained from four groups. Data were presented as mean ± SD (standard deviation). The One-way Analysis of variance (ANOVA) followed by Tukey's posthoc test (for normally distributed data) or Kruskal Wallis nonparametric ANOVA followed by Dunn's multiple comparisons test (for the data which were not in a normal distribution) was administrated to find out significant differences between the groups. A Pearson or Spearman correlation coefficient was used to determine the correlation between two variables. The direct or inverse correlation was indicated by a positive or negative correlation coefficient (r) value, respectively. The distribution of categorical variables like gender distributions among study groups was presented as percentage (%) and compared by the Chi-Square test. A value of p < 0.05 was considered statistically significant. The statistical analysis was performed using Graph Pad Prism software (version 5, 2007, San Diego, California, United States).

## Results

Comparison of demographic parameters among study groups:

As shown in the Table 2, different study groups enrolled in the present study showed no statistically significant differences for age, gender distributions, body mass index (BMI), duration of diabetes, and blood pressure. Glycaemic parameters like FPG, PPG and HbA1C levels also demonstrated no significant differences among diabetic subgroups like DNR, MNPDR and HRPDR.

**Table 1:** Number of subjects with indications for vitrectomy in each sub-group of the study

Indications	Study sub-groups			
	HC	DNR	MNPDR	HRPDR
Idiopathic macular hole	9	18	19	–
Removal of an accidentally dropped nucleus after blunt trauma	3	2	2	–
Per-operative complications of phacoemulsification	2	2	3	–
Vitreous haemorrhage	–	–	–	24

HC = healthy control (non-diabetic); DNR = diabetics with no retinopathy; MNPDR = mild non-proliferative diabetic retinopathy; HRPDR = high risk proliferative diabetic retinopathy

**Table 2:** Demographic and clinical parameters of study subjects

Parameters	HC (N = 14)	DNR (N = 22)	MNPDR (N = 24)	HRPDR (N = 24)	p value
Age (years)	51.00 ± 7.48	51.02 ± 6.12	53.11 ± 1.99	52.06 ± 6.61	0.649
Gender					
M	8 (57.14%)	12 (54.54%)	12 (50%)	13 (54.16%)	0.976
F	6 (42.85%)	10 (45.45%)	12 (50%)	11 (45.83%)	
BMI (kg/m^2^)	23.36 ± 3.412	25.17 ± 2.314	25.87 ± 3.972	24.02 ± 6.624	0.334
Duration of DM (years)	–	10.46 ± 5.191	11.14 ± 3.916	11.36 ± 3.684	0.784
Blood Pressure (mm Hg)					
Systolic	123.2 ± 9.575	127.8 ± 6.012	127.6 ± 2.914	126.3 ± 4.214	0.116
Diastolic	80.71 ± 3.286	82.39 ± 4.591	80.27 ± 4.010	81.32 ± 5.113	0.435
Glycaemic Status					
FPG (mg/dl)	80.40 ± 8.444	151.9 ± 9.52****	158.6 ± 27.36^a^	154.7 ± 16.11!!!!	< 0.0001
PPG (mg/dl)	118.2 ± 10.16	181.22 ± 32.90 ***	194.67 ± 61.32^a^	210.64 ± 29.92!!!!	< 0.0001
HbA1C (%)	4.76 ± 0.2881	7.91 ± 0.412****	8.48 ± 1.10^a^	8.1 ± 0.98!!!!	< 0.0001
Lipid profile					
TCH (mg/dl)	155.0 ± 8.337	184.6 ± 28.11	211.71 ± 25.17^b^	200.71 ± 28.62!!	0.005
LDL-C (mg/dl)	93.40 ± 21.14	115.2 ± 23.2	136.61 ± 17.84	130.43 ± 25.89!!	0.013
HDL-C (mg/dl)	45.40 ± 7.232	42.21 ± 7.31	40.81 ± 5.990	41.02 ± 3.262	0.412
TG (mg/dl)	100.6 ± 29.95	143.44 ± 19.23	161.14 ± 22.89^c^	191.22 ± 40.89!!,§§	< 0.0001

HC, healthy control; DNR, diabetic subjects without clinically evident retinopathy, MNPDR, early non-proliferative diabetic retinopathy without macular edema; HRPDR, high-risk proliferative diabetic retinopathy; BMI, body mass index; FPG, fasting plasma glucose, PPG, postprandial plasma glucose; HbA1c, glycatedhemoglobin; TCH, total cholesterol; LDL-C, low density lipoprotein cholesterol, HDL-C, high density lipoprotein cholesterol; TG, triglycerides. The Kruskal Wallis nonparametric ANOVA followed by Dunn's multiple comparisons test was administrated to find out significant differences between the groups. A value of p < 0.05 was considered as statistically significant.

^****^, *** indicates HC vs DNR, p < 0.0001 and < 0.001 respectively

a, b, c indicates HC vs MNPDR, p < 0001, p < 0.001 and p < 0.01 respectively

!!!!, !! Indicates HC vs HRPDR, and p < 0001respectively

^§§^ indicates DNR vs HRPDR, p < 0.01

### Comparison of conventional lipid parameters, TL and FFA among study groups

- Regarding plasma TCH, both the MNPDR and HRPDR subjects showed higher TCH levels than HC subjects. The study showed no significant variation in TCH level among DNR, MNPDR, and HRPDR respectively.
- The HRPDR subject showed a higher LDL-C level compared to HC. However, the study showed no significant differences when plasma LDL-C level was compared among HC, DNR, and MNPDR respectively. The HDL-C level also demonstrated no significant differences between the study groups.
- The TG levels of MNPDR and HRPDR subjects were found to be higher when compared with HC subjects. The HRPDR subjects also showed higher TG levels compared to DNR subjects. In contrast, the TG level of MNPDR subjects showed no significant alteration when compared with DNR and HRPDR subjects (Table 2).
- The concentration of TL and FFA in plasma and vitreous were found to be significantly elevated stepwise inT2DM subjects (HRPDR > MNPDR > DNR) compared to HC (Table 3).

### Comparison of lipid peroxides and ALE among study groups

The plasma and vitreous levels of LPOs like MDA and HNE, and ALE like HLY were found to be significantly higher among DNR, MNPDR, and HRPDR subjects compared to HC subjects. Again, both the MNPDR and HRPDR subjects showed a significantly higher level of LPO and ALE components compared to DNRs. Further, the HRPDR subjects showed higher levels of the same parameters than the MNPDRs (Tables 3 and 4).

**Table 3:** Plasma level of different lipid components (TL, FFA), lipid peroxides (MDA, HNE), advanced lipoxidation end products (ALEs), and endothelial dysfunction marker (VEGF) among study groups

Parameters	HC (N = 14)	DNR (N = 22)	MNPDR (N = 24)	HRPDR (N = 24)	p value
TL (mg/dl)	550.76 ± 32.26	695.69 ± 100.51**	780.62 ± 99.22††, ¥	892.34 ± 99.24!!!!, §§§§, ‡‡	< 0.0001
FFA (mmol/L)	0.308 ± 0.015	0.618 ± 0.014****	0.741 ± 0.059††††, ¥¥¥¥	0.792 ± 0.024!!!!, §§§§, ‡	< 0.0001
MDA (µmol/L)	5.19 ± 0.968	8.02 ± 0.842***	11.70 ± 0.282††††, ¥¥¥¥	15.25 ± 0.991!!!!,§§§§, ‡‡‡	< 0.0001
HNE (pg/ml)	8664 ± 3016	14,070 ± 3556****	19,339 ± 3184††††,¥¥¥	27,290 ± 1252!!!!,§§§§, ‡‡‡‡	< 0.0001
HLY (nmol/L)	89.50 ± 16.71	187.62 ± 20.11****	276.32 ± 40.01††††, ¥¥¥¥	359.48 ± 34.62!!!!,§§§§, ‡‡‡‡	< 0.0001
VEGF (pg/L)	72.21 ± 7.695	91.10 ± 10.34*	129.04 ± 20.22††††, ¥¥¥¥	226.16 ± 19.84 !!!!, §§§§, ‡‡‡‡	< 0.0001

The The one-way analysis of variance (ANOVA) followed by Tukey's test and Kruskal Wallis test nonparametric ANOVA followed by Dunn's multiple comparisons test was administrated to find out significant differences between the groups. A value of p < 0.05 was considered as statistically significant

TL, total lipid; FFA, free fatty acid; HNE, 4-Hydroxynonenal; MDA, malondialdehyde; HLY, hexanoyl-lysine; VEGF, vascular endothelial growth factor.

HC vs DNR, * = p < 0.05; ** = p < 0.01; *** = p < 0.001, **** = p < 0.0001

HC vs MNPDR, †† = p < 0.01; †††† = p < 0.0001

HC vs HRPDR, !!!! = p < 0.0001

DNR vs MNPDR, ¥ = p < 0.05;¥¥¥ = p < 0.001; ¥¥¥¥ = p < 0.0001

DNR vs HRPDR, §§§§ = p < 0.0001

MNPDR vs HRPDR, ‡ = p < 0.05, ‡‡ = p < 0.01, ‡‡‡ = p < 0.001; ‡‡‡‡ = p < 0.0001

**Table 4:** Vitreous level of different lipid components (TL, FFA), lipid peroxides (MDA, HNE), advanced lipoxidation end products (ALEs), and endothelial damage marker (VEGF) among study groups

Parameters	HC (N = 14)	DNR (N = 22)	MNPDR (N = 24)	HRPDR (N = 24)	p value
TL (mg/dl)	68 ± 20.24	132.68 ± 19.89***	199.22 ± 22.48††††, ¥¥¥¥	228.52 ± 29.16!!!!,§§, ‡	< 0.0001
FFA (mmol/L)	0.0760 ± 0.017	0.1475 ± 0.036*	0.1982 ± 0.064†††,¥	0.2444 ± 0.0211!!!!, §, ‡	< 0.0001
MDA (µmol/L)	1.19 ± 0.5348	2.74 ± 0.366*	4.49 ± 0.613††††, ¥¥	7.97 ± 1.672!!!!,§§§§, ‡‡‡‡	< 0.0001
HNE (pg/ml)	2132 ± 1223	4211 ± 949.2****	5404 ± 783.11††††,¥¥	7368 ± 5877!!!!,§§§§, ‡‡‡‡	< 0.0001
HLY (nmol/L)	75.98 ± 12.97	84.90 ± 16.90*	106.11 ± 5.62†††,¥	126.4 ± 3.09!!!!, §§§§, ‡	< 0.0001
VEGF (pg/L)	72.06 ± 5.109	90.50 ± 6.585*	124.24 ± 10.14††††, ¥¥¥¥	134.46 ± 14.21!!!!, §§§§, ‡	< 0.0001

The The one-way analysis of variance (ANOVA) followed by Tukey's test and Kruskal Wallis test nonparametric ANOVA followed by Dunn's multiple comparisons test was administrated to find out significant differences between the groups. A value of p < 0.05 was considered as statistically significant

TL, total lipid; FFA, free fatty acid; HNE, 4-Hydroxynonenal; MDA, malondialdehyde; HLY, hexanoyl-lysine; VEGF, vascular endothelial growth factor

HC vs DNR, * = p < 0.05; *** = p < 0.001, **** = p < 0.0001

HC vs MNPDR, ††† = p < 0.001; †††† = p < 0.0001

HC vs HRPDR, !!!! = p < 0.0001

DNR vs MNPDR, ¥ = p < 0.05; ¥¥ = p < 0.01; ¥¥¥¥ = p < 0.0001

DNR vs HRPDR, § = p < 0.05; §§ = p < 0.01, §§§§ = p < 0.0001

MNPDR vs HRPDR, ‡ = p < 0.05; ‡‡‡‡ = p < 0.0001

### Comparison of VEGF level among study groups

The endothelial dysfunction induced secretion of VEGF level both in the plasma and vitreous demonstrated higher values in the diabetic subgroups like DNR, MNPDR, and HRPDR subjects compared to HC subjects. Again, both the MNPDR and HRPDR subjects showed a significantly higher level of VEGF than DNRs. Further, the HRPDR subjects showed a higher VEGF level in plasma and vitreous than the MNPDR ones (Tables 3 and 4).

### Correlation between plasma and vitreous level of lipid components in HC and T2DM (subjects from DNR + MNPDR + HRPDR) group

In the case of T2DM subjects, the plasma level of TL, FFA, LPOs, ALE components, and VEGF showed strong and significant positive correlations with their levels in vitreous. However, the study showed no significant correlation between plasma and vitreous levels of those lipid components among HC individuals (Table 5).

**Table 5:** Correlation between plasma level of lipid components and VEGF with their levels in vitreous among HC and T2DM (subjects from DNR + MNPDR + HRPDR) group

Parameters	HC	T2DM
TL (mg/dl)	r = 0.35, p = 0.47	r = 0.76, p < 0.0001
FFA (mmol/L)	r = 0.21, p = 0.64	r = 0.67, p < 0.0001
MDA (µmol/L)	r = 0.31, p = 0.54	r = 0.84, p < 0.0001
HNE (pg/ml)	r = 0.32, p = 0.49	r = 0.79, p < 0.0001
HLY (nmol/L)	r = 0.29, p = 0.56	r = 0.73, p < 0.0001
VEGF (pg/L)	r = 0.28, p = 0.60	r = 0.72, p < 0.0001

The DNR, MNPDR and HRPDR groups are combined together and termed as Type 2 Diabetes Mellitus (T2DM) group. Pearson or Spearman's rank correlation coefficient (r) was used and a value of p < 0.05 was considered as statistically significant

TL, total lipid; FFA, free fatty acid; HNE, 4-Hydroxynonenal; MDA, malondialdehyde; HLY, hexanoyl-lysine; VEGF, vascular endothelial growth factor

### Correlation of plasma level of conventional lipid components, TL and FFA with vitreous level of LPOs, ALE and VEGF in DNR and DR (MNPDR + HRPDR) individuals

In the case of DNR subjects, the plasma level of TCH, LDL-C, TG, TL, and FFA showed significant positive correlations with vitreous levels of LPOs, ALE components, and VEGF (Table 6). However, the study showed no significant correlations among the same in the DNR group (Table 7).

**Table 6:** Correlation of plasma level of conventional lipid components, TL and FFA with vitreous level of LPOs, ALE and VEGF in DNR subjects

Parameters	MDA (µmol/L)	HNE (pg/ml)	HLY (nmol/L)	VEGF (pg/L)
TCH (mg/dl)	r = 0.243, p = 0.152	r = 0.391, p = 0.092	r = 0.369, p = 0.081	r = 0.379, p = 0.087
LDL-C (mg/dl)	r = 0.25, p = 0.187	r = 0.368, p = 0.119	r = 0.385, p = 0.078	r = 0.294, p = 0.196
HDL-C (mg/dl)	r = 0.094, p = 0.416	r = 0.148, p = 0.491	r = 0.094, p = 0.625	r = 0.224, p = 0.439
TG (mg/dl)	r = 0.234, p = 0.148	r = 0.384, p = 0.074	r = 0.363, p = 0.080	r = 0.432, p = 0.071
TL (mg/dl)	r = 0.392, p = 0.079	r = 0.351, p = 0.079	r = 0.381, p = 0.076	r = 0.382, p = 0.081
FFA (mmol/L)	r = 0.366, p = 0.077	r = 0.371, p = 0.104	r = 0.316, p = 0.154	r = 0.379, p = 0.075

The DNR, MNPDR and HRPDR groups are combined together and termed as Type 2 Diabetes Mellitus (T2DM) group. Pearson or Spearman's rank correlation coefficient (r) was used and a value of p < 0.05 was considered as statistically significant

TCH, total cholesterol; LDL-C, low density lipoprotein cholesterol, HDL-C, high density lipoprotein cholesterol; TG, triglycerides; TL, total lipid; FFA, free fatty acid; HNE, 4-Hydroxynonenal; MDA, malondialdehyde; HLY, hexanoyl-lysine; VEGF, vascular endothelial growth factor

**Table 7:** Correlation of plasma level of conventional lipid components, TL and FFA with vitreous level of LPOs and ALE and VEGF in DR (MNPDR + HRPDR) subjects

Parameters	MDA(µmol/L)	HNE(pg/ml)	HLY (nmol/L)	VEGF (pg/L)
TCH (mg/dl)	r = 0.542, p = 0.010	r = 0.451, p = 0.026	r = 0.431, p = 0.024	r = 0.524, p = 0.014
LDL-C (mg/dl)	r = 0.512, p = 0.016	r = 0.592, p = 0.002	r = 0.552, p = 0.012	r = 0.439, p = 0.028
HDL-C (mg/dl)	r = 0.136, p = 0.415	r = 0.174, p = 0.395	r = 0.161, p = 0.423	r = 0.191, p = 0.381
TG (mg/dl)	r = 0.693, p < 0.0001	r = 0.586, p = 0.012	r = 0.541, p = 0.023	r = 0.536, p = 0.011
TL (mg/dl)	r = 0.752, p < 0.0001	r = 0.481, p = 0.018	r = 0.591, p = 0.002	r = 0.616, p = 0.001
FFA (mmol/L)	r = 0.610, p = 0.001	r = 0.475, p = 0.024	r = 0.691, p < 0.0001	r = 0.453, p = 0.026

The DNR, MNPDR and HRPDR groups are combined together and termed as Type 2 Diabetes Mellitus (T2DM) group. Pearson or Spearman's rank correlation coefficient (r) was used and a value of p < 0.05 was considered as statistically significant

TCH, total cholesterol; LDL-C, low density lipoprotein cholesterol, HDL-C, high density lipoprotein cholesterol; TG, triglycerides; TL, total lipid; FFA, free fatty acid; HNE, 4-Hydroxynonenal; MDA, malondialdehyde; HLY, hexanoyl-lysine; VEGF, vascular endothelial growth factor

## Discussion

Hyperglycemia associated with dyslipidemia has received much more attention in recent years and is also being considered to be a strong risk factor for the development of DR [26, 27]. In dyslipidemia, higher serum lipid levels cause retinal endothelial dysfunction by reducing the bioavailability of nitric oxide (NO), which is suggested to accelerate DR pathogenesis [26]. However, clinical studies demonstrated discrepancies and inconclusive results regarding the association of serum lipids with the pathogenesis and severity of DR. The ETDRS and Wisconsin epidemiology study of DR showed a statistically significant association of serum TCH and LDL levels with the severity of retinal hard exudation in patients with DR. [28]. The Chennai Urban Rural Epidemiology study found a higher serum lipid among subjects with DR than DNR [8]. The EURODIAB IDDM Complications study group showed that TCH is related to all levels of DR and the elevated serum TG is associated with moderately severe NPDR and PDR [29]. The therapeutic use of lipid-lowering drugs such as fibrates and cholesterol-lowering drugs such as statins showed favourable effect for DR [30, 31]. However, the other larger studies such as the Multi-Ethnic Study of atherosclerosis and Australian Diabetes Obesity and lifestyle study did not confirm those findings [32, 33]. In addition, the ‘Singapore Malay Eye Study’ reported that the higher TCH level protects from the pathogenesis of DR [34].

In the present study, plasma conventional lipid components of diabetic subjects like TCH, LDL-C showed a tendency of gradually increasing towards the pathogenesis of DR, and TG was found to be increased towards pathogenesis with the severity of DR. The findings are partially supported by Cowdhuri et al., 2017 and Pramanik et al., 2020 who have demonstrated a significant increment of TCH, LDL-C, and TG levels in MNPDR compared to HC and DNR [1, 22]. Another prospective study by Prajapati et al., 2017 showed a stepwise increment of TCH, LDL-C, and TG from NPDR to PDR [35]. However, there is lack of evidences regarding the mechanism by which plasma conventional lipid components cause DR. An animal model study revealed that, higher TCH level impairs retinal function by diminishing retinal ganglion cell density, decreasing thickness of the photoreceptor layer and inner nuclear layer by increasing of inducible nitric oxide synthase (iNOS) activity [36]. Another rabbit model study showed that high cholesterol level increases a peptide named amyloid-beta (Aβ), in retinal photoreceptors, that increases ROS production, decreases peroxide levels and there by damages neuronal layer of diabetic retina [30]. In diabetic condition TCH is also auto-oxidised and produce 7-ketocholesterol (7kCh) is a potent pro-apoptotic agent that activates caspase and turns apoptosis of the retinal cells and may expedite the pathogenesis and severity of DR [37]. Under higher LDL-C level in diabetes, increased LDL-C uptake through retinal pigment epithelial (RPE) layer causes increased permeability of retinal capillaries and extravasation of plasmalipoproteins. On the other hand, oxidized and glycated LDL-C identified in the retina during diabetes were shown to induce retinal pericyte loss and that is considered to play an important role in diabetic retinopathy [38].

The blood level of higher TG incorporated into the cell membrane alters membrane fluidity, permeability and leads to haemorrhage and oedema. It also triggers local inflammatory response releasing cytokines, growth factors that are responsible for retinal endothelial dysfunction, neovascularization and may also facilitate the pathogenesis and severity of DR [39].

Plasma TL and FFA were found to be gradually elevated in concentration in both plasma and vitreous starting from DNR to HRPDR.A study by Salaria and Vyas, 2019 have reported that the overall elevation of plasma lipid levels is associated with endothelial dysfunction, which appears to play an important role in the pathogenesis of DR, particularly with the breakdown of the blood-retinal barrier [40]. Hence, there is very low evidenceregarding the association of higher FFA with DR pathogenesis. Researchers have demonstrated that the excess FFA level is associated with lipo-toxicity [41]. The di-acyl glycerol (DAG) and ceramide, collectively formed due to the esterification of sphingosine with long chain saturated FFAs contribute to the development of endoplasmic reticulum (ER) stress, mitochondrial dysfunction and the generation of ROS, which together result in inflammation, insulin resistance and apoptosis [42, 43]. Some studies have shown that the activity of some glycolytic enzyme-like Hexokinase (HK), glucokinase, phosphofructokinase, Glucose-6-phosphate dehydrogenase (G6PDH), 6 phosphogluconate dehydrogenase are badly affected by FFA octanoate [44, 45]. So, it may be hypothesized that all these said events converge and probably play an essential role in DR pathogenesis and progression of DR.

The present study also demonstrated a gradual increment of LPO products like MDA and HNE and ALE like HLY both in plasma and vitreous sample which might be associated with the development and progression of DR. The studies by Chatziralli et al., 2017, and Mondal et al., 2021 showed increased plasma MDA level aggravates DR pathogenesis [46, 47]. Another study by Mancino et al., 2017 revealed increased vitreous MDA level among NPDR and PDR subjects compared to non-diabetic HC subjects [48]. Researchers have demonstrated that the MDA compound is associated with protein modification in a pH dependent fashion [49]. Likewise, different clinical studies have demonstrated elevated circulatory levels of lipid peroxidation products like HNE in diabetic patients with DR in comparison to DNR and HCs, whereas animal studies also confirmed these findings by documenting increased concentration of HNE and HNE-derived advanced lipoxidation end products in diabetic rats [49, 50]. Similarly some investigators revealed the contribution of HNE in the pathogenesis of DR by activating the Wingless-related integration site (WNT) signaling pathway, whereas some experimental studies revealed the role of HNE in retinal perfusion deficit and hemodynamic changes owing to reduced activity of large-conductance Ca^2+−^activated K^+^ channel (BK channel) on retinal vascular smooth muscle cells [51, 52]. Another experiment in cell-culture media containing human retinal capillary pericyte and Muller glia exposed to HNE illustrated the development of endoplasmic reticulum stress, mitochondrial dysfunction, and apoptosis of cells [53].

### The graded level of increment of ALE components like HLY from HC to HRPDR

(HC < DNR < MNPDR < HRPDR) was also found to be increased in the present study. A previous study by Chowdhuri et al., 2017 reported a significant elevation of ALE levels among DNR and MNPDR subjects compared to HCs. Moreover, significant elevation of HLY in the vitreous and serum of patients with.

### PDR was also observed by Izuta et al., 2010, which was following our findings [1, 54]

The present study also showed significant correlations of plasma level TL, FFA, LPO, ALE components, and VEGF with their level in vitreous of the T2DM subjects. This finding indicated that the abnormality of systemic lipid homeostasis and endothelial dysfunction induced secretion of VEGF that occurs in diabetic conditions is similarly extended to retinal microvascular tissues also. Additionally, plasma conventional lipid components (like TCH, LDL-C, and TG), TL, and FFA showed significant correlations with vitreous LPO and ALE products and VEGF in the DR group in the present study. This finding suggests that DR is not only associated with a gradual rise of lipid levels but also lipid peroxidation reactions and ALE product formation aggravated and that may further accelerate its severity by augmenting endothelial dysfunction. Recently researchers have also demonstrated that lipoxidation is closely linked with oxidative stress which results the activation of several cell death pathways such as apoptosis and necrosis [55]. Endothelial dysfunction in the pathogenesis of DR is characterized by an imbalance between endothelium-derived vasodilator and vasoconstrictor substances like endothelin and inducible NO. Occlusion of retinal (and elsewhere) small blood vessels and consequent ischemia trigger up-regulation of angiogenic factors (VEGF) and inflammatory mediators, which induce increase in capillary permeability and angiogenesis in order to overcome and bypass the occluded small blood vessels [56]. So, the products of lipoxidation contribute to endothelial dysfunction of capillary beds of retina which may stimulate increased secretion of VEGF and manifestation of DR.

### Conclusion

This study concludes that the association of lipid-derived toxic metabolites due to increased lipid peroxidation and lipoxidation in a hyperglycemic environment may be responsible for the development and severity of DR. Determination of concentration of lipid-derived toxic molecules and VEGF in blood and vitreous of diabetic patients with different stages of retinopathy in this study, definitely reflect strong correlation of lipid peroxidation and stimulation of excessive VEGF secretion in retinal tissue. To understand better the pathochemistry of DR, more prospective large-scale studies would be needed to elucidate the association between different lipid derivatives and stages of DR.

## Data Availability

The data that support the findings of this study are available from the corresponding author, [LKM], upon reasonable request.
